# Individualized treatment of HER2-positive breast cancer patients with lung metastasis using a circulating tumor cell drug sensitivity test: a case report

**DOI:** 10.3389/fonc.2026.1888730

**Published:** 2026-09-25

**Authors:** Yangyang Qian, Taihen Yu, Miaochun Zhong, Wenjie Xia, Haibo Zhang, Mingxiang Kong

**Affiliations:** 1 General Surgery, Cancer center, Department of Breast Surgery, Zhejiang Provincial People’s Hospital, Affiliated People’s Hospital, Hangzhou Medical College, Hangzhou, Zhejiang, China; 2 Cancer Center, Department of Radiology, Zhejiang Provincial People’s Hospital, Affiliated People’s Hospital, Hangzhou Medical College, Hangzhou, Zhejiang, China; 3 Center for Rehabilitation Medicine, Department of Orthopedics, Zhejiang Provincial People’s Hospital, Affiliated People’s Hospital, Hangzhou Medical College, Hangzhou, Zhejiang, China

**Keywords:** breast cancer, case report, circulating tumor cell, drug testing, individualized treatment

## Abstract

Breast cancer is a leading cause of tumor-related death in women worldwide. Despite treatment advances, survival from metastatic breast cancer is still not satisfactory. Therefore, timely detection of effective treatment is crucial. Changes in receptor status have been reported between primary tumors and metastases. These were mainly due to the changes in the molecular profiles of breast cancer and the heterogeneity within the tumor. Recent studies have suggested that circulating tumor cells (CTCs) can aid prognostic monitoring and guide individualized treatment by assessing chemotherapy sensitivity. Here, we described two cases of HER2-positive breast cancer patients with lung metastasis using CTC drug sensitivity test for individualized treatment. The results suggest that CTC drug sensitivity test demonstrates good value in guiding individualized treatment. Furthermore, the CTC drug sensitivity test results were found to align well with the histopathological results and the patients’ prior tumor resistance information. These observations preliminarily showcase the potential of CTCs to predict metastatic lesions and to identify effective treatments, especially when histopathology is unavailable. In summary, our case report is the first to evaluate CTC drug sensitivity testing in advanced breast cancer, showing its clinical value and feasibility for personalized therapy, and suggests its broad development prospects in the future. However, further research is needed to validate its reliability.

## Introduction

Breast cancer is one of the leading causes of tumor-related death in women worldwide (1). Despite advances in treatments, survival from metastatic breast cancer is still not satisfactory, with an overall survival (OS) of approximately 31% (2). Therefore, timely detection of effective treatment is crucial. Breast cancer tends to metastasize to target organs, such as the lung, bone, liver, and brain (3). For patients with metastatic breast cancer, 21%–32% have lesions in the lung, 15%–32% in the liver, 30%–60% in the bone, and 4%–10% in the brain (4). In addition, breast cancer can be classified into different subgroups based on their gene expression profiles, and different tumor subtypes tend to metastasize to distinct target organs (5). For instance, the luminal subtype tends to metastasize to the bone, while the basal-like subtype shows a propensity for lung metastasis (5). Important regulators of breast cancer dissemination mainly include cancer stem cells (CSCs) and relevant signaling pathways, chemokines, and the tumor microenvironment. CSCs, also known as tumor-initiating cells, have been reported to have the ability to self-renew and differentiate and to be able to recapitulate the heterogeneity of tumors (5–7). Changes in the receptor status have been reported by a number of articles between primary tumors and metastases, and the rates of altered receptor status were approximately 20%–40% for progesterone receptor (PR), 20%–36% for estrogen receptor (ER), and 10%–20% for human epidermal growth factor receptor 2 (HER2) (8, 9). These changes were mainly due to the changes in the molecular profiles of breast cancer and the heterogeneity within the tumor (10).

Liquid biopsy is a newly developed diagnostic and treatment technology with good prospects, mainly including circulating tumor cells (CTCs), circulating tumor DNA (ctDNA), and exosomes (11). CTCs are tumor cells isolated from the primary tumor site and transported through the circulatory system. They could spread to the distal end of the body, forming metastatic lesions and regenerating secondary tumors before eventually leaking out or becoming lodged in the capillaries in certain organs (12, 13). CTCs could play a significant role in breast cancer metastasis by improving the adaptive mechanism for their survival in the microenvironment of the circulatory system (13). Recently, some studies have suggested that CTCs could be used for the early diagnosis and prognostic monitoring of tumors (14–16) and may provide individualized treatment information by evaluating the sensitivity of tumor cells to chemotherapy drugs (17, 18). Here, we described two cases of HER2-positive breast cancer patients with lung metastasis identified using the CTC drug sensitivity test to guide individualized treatment.

## Methods

In our study, on the basis of removing CD45^+^ cells from the blood with magnetic cell sorting (MACS), we also simultaneously labeled for the epithelial cell marker EpCAM (epithelial cell adhesion molecule) and the mesenchymal cell marker cell surface vimentin (CSV) to improve the rate of CTC acquisition (28). To identify the optimum therapy for individualized treatment, a personalized CTC drug testing system was conducted. Thousands of CTCs were seeded into a multi-well chip and subjected to differential drug treatment evaluations. It was confirmed that the gene expression profiles across different wells exhibited a high degree of correlation, and the CTC gene expression was consistent with that of the original tumor.

In clinical practice, the Response Evaluation Criteria in Solid Tumors (RECIST) are commonly used to evaluate the effectiveness of individualized treatment (29). Firstly, we determined the CTC cutoff value based on the RECIST, which used 0.7 as a cutoff value, and defined complete response (CR) or partial response as effective and stable disease (SD) or progressive disease (PD) as ineffective (30). The cell viability after dosing a drug A was defined with the following formula: *p*_A_ = *R*_A_/*R*_0_, where *R*_A_ represents the living rate of CTCs in the drug A group and *R*_0_ represents the living rate of CTCs in the control group. If *p*_A_ < 0.7, then it was considered effective; otherwise, it was ineffective.

After determining the cutoff as 0.7, we used the CTC samples of 12 patients as the training cohort to determine the effective drug concentration values. Three replications were performed for each drug sample pair. We determined the optimal drug concentration by adjusting the concentration of drug A so that the overall kill rate of the drug was close to the overall response rate in related clinical trials. In the drug sensitivity test, enriched CTCs should be ≥20/ml and were directly treated with drugs *in vitro* for 24 h. Due to the varying exposure times of the different drugs, the drug exposure time has been uniformly set at 24 h to ensure adequate and uniform exposure. Subsequently, the CTCs (5 × 10^3^/well) were suspended in RPMI-1640 medium and inoculated into 96-well plates. Dimethyl sulfoxide (DMSO, control) and various anticancer drugs were added, along with three commonly used green fluorescent-labeled antibodies against CTC biomarkers (i.e., anti-EpCAM, anti-CSV, and anti-PD-L1), which were added to the culture system simultaneously. After incubation for 30–60 min, propidium iodide (PI) was added to detect dead CTCs. Images were captured and cells were manually enumerated.

## Case presentation

### Case 1

A 56-year-old woman presented with a 3-cm × 2-cm mass in the right breast. Preoperative biopsy revealed invasive ductal carcinoma of the right breast [immunohistochemistry: ER(−), PR(−), HER2(3+), Ki67(+35%)], and axillary lymph nodes were negative. Preoperative evaluation showed no signs of metastasis in all organs (including the lungs). Therefore, the patient underwent right modified radical mastectomy with sentinel lymph node biopsy. The final pathology revealed a 2.5-cm × 2.0-cm invasive ductal carcinoma of the right breast [immunohistochemistry: ER(−), PR(−), HER2(3+), Ki67(+70%)], with negative sentinel lymph nodes (0/5). After 1 month, a computed tomography (CT) scan showed multiple lung nodules that were not detected by preoperative CT and were considered metastatic (**Figure 1A**). Her final TNM stage was T2N0M1. Thus, therapy with the PTH regimen [docetaxel 100 mg/m^2^ (day 1, d1), trastuzumab 8 mg/kg (d1) followed by 6 mg/kg (d1), and pertuzumab 840 mg (d1) followed by 420 mg (d1), 1/21d] was chosen according to the guidelines. Nevertheless, pulmonary nodules gradually grew larger and more numerous after four cycles of treatment (**Figure 1B**). Positron emission tomography (PET)/CT revealed multiple metastatic nodules in both lungs, with the largest being approximately 9 mm × 7 mm (**Supplementary Figure S1A**). Therefore, we implemented a CTC drug sensitivity test to screen for effective drugs for individualized treatment. All drugs used for the CTC sensitivity test were selected based on the guidelines and the clinical experience of physicians. Interestingly, the CTC sensitivity test results revealed similar tumor cell lethality between chemotherapy alone and chemo-targeted combinations (**Figure 2**; **Supplementary Figure S2**). This discovery indicates that the previously used treatment regimen (the PTH regimen) was ineffective. At the same time, pulmonary nodules were punctured, and the histopathological diagnosis was consistent with lung metastasis from breast cancer [immunohistochemistry: ER(−), PR(−), HER2(1+), Ki67(+30%)] (**Supplementary Figure S4**). This result suggested a change in the HER2 status of the patient, which was positive in the primary tumor and negative in the metastatic pulmonary nodules. This was consistent with our test results and may explain why targeted therapy based on the CTC sensitivity testing was ineffective. Finally, based on the CTC drug sensitivity screening results, the NCb [vinorelbine 25 mg/m^2^ (d1, d8) and carboplatin area under the curve (AUC) = 2 (d1, d8), 1/21d] plus a targeted therapy [trastuzumab 6 mg/kg (d1) and pertuzumab 420 mg (d1), 1/21d] regimen was selected for the patient. After four cycles of treatment, the pulmonary nodules almost completely disappeared (**Figure 1C**; **Supplementary Figure S1B**). At present, the patient has reached progression-free survival (PFS) for 14 months. A comprehensive overview of the disease progression and treatment regimen is depicted in **Supplementary Figure S5**.

**Figure 1:**
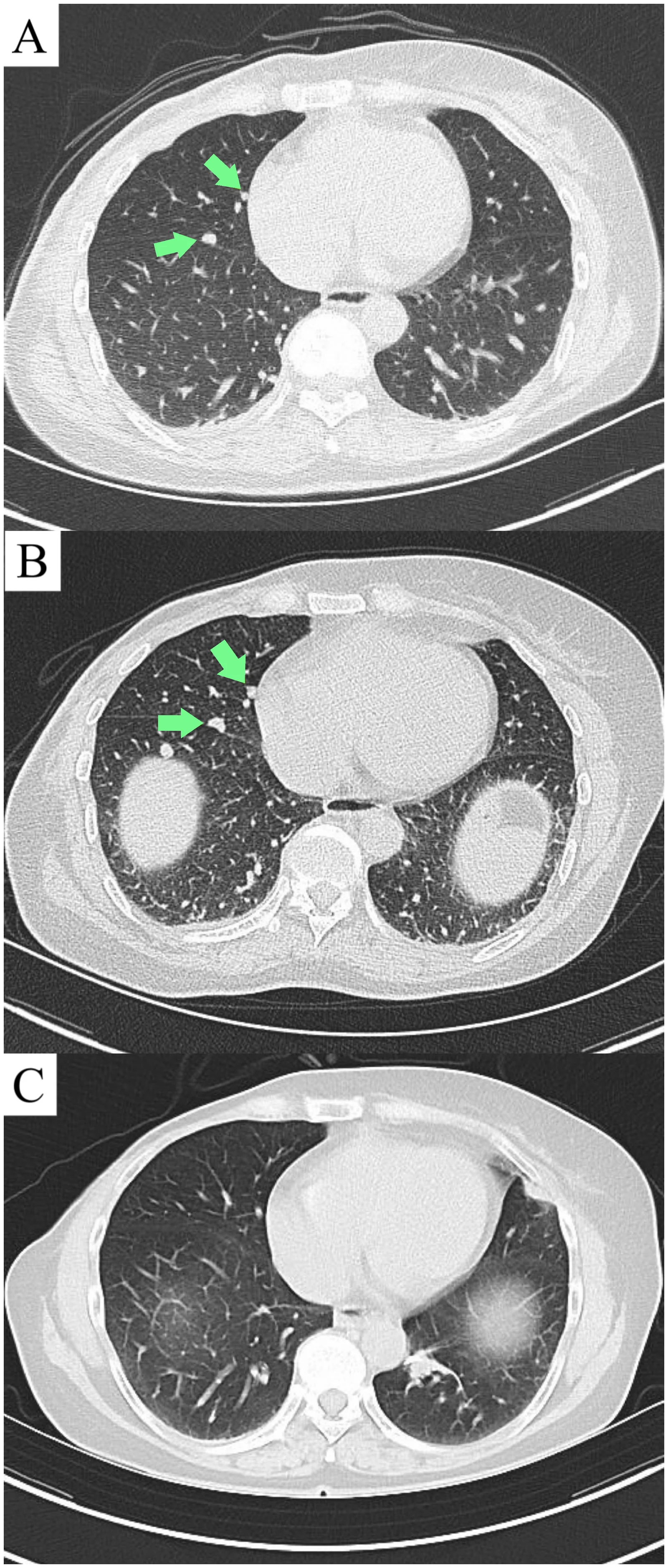
**(A)** Axial CT lung window demonstrating multiple pulmonary nodules, which was considered pulmonary metastasis. **(B)** Axial CT lung window demonstrating pulmonary nodules were markedly enlarged after four cycles of PTH treatment. **(C)** Axial CT lung window demonstrating that pulmonary nodules almost disappeared after four cycles of NCbPH treatment.

**Figure 2:**
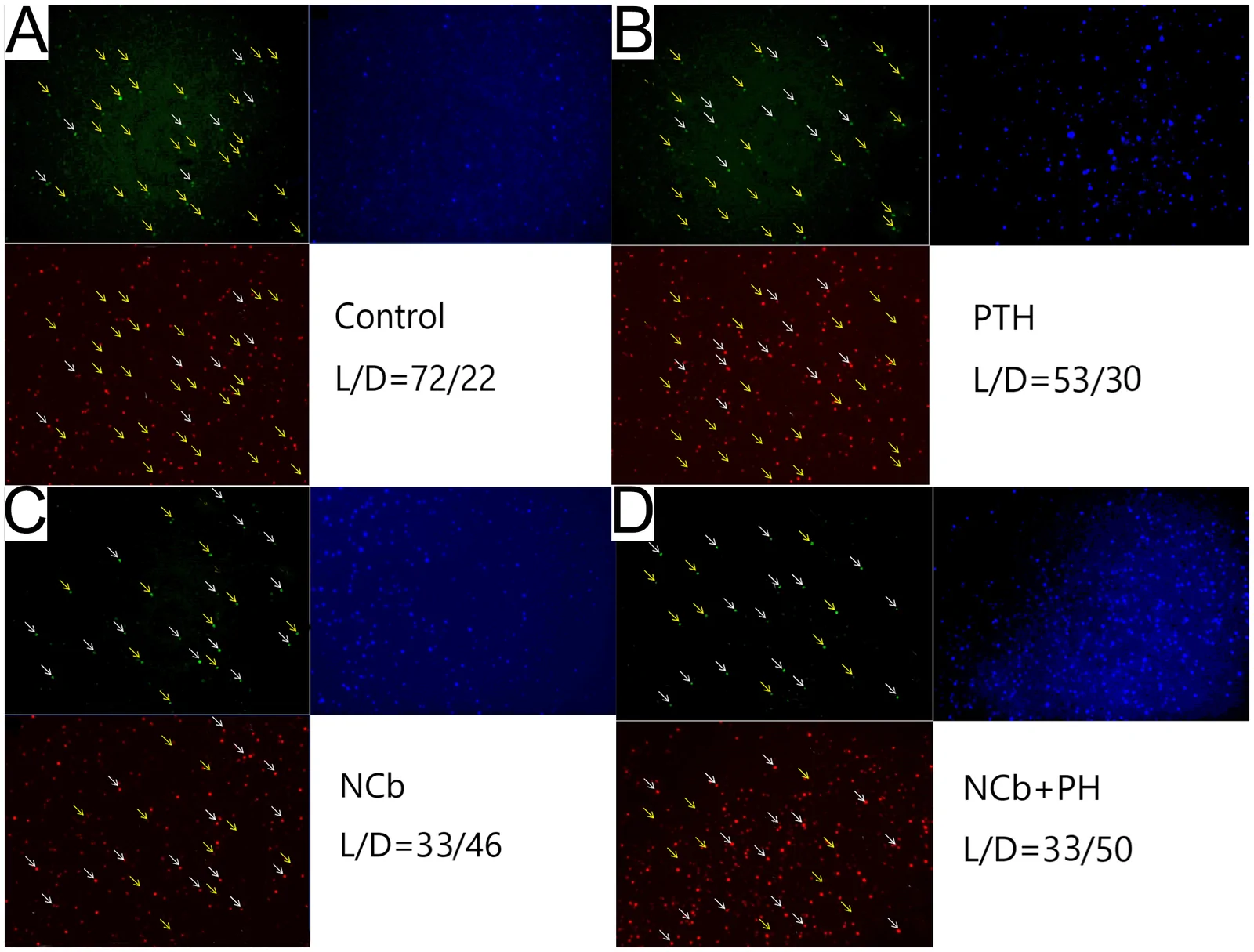
CTC drug sensitivity test results for individualized treatment in case 1. Green fluorescence indicates CTCs, and red fluorescence indicates dead cells. The yellow arrows represent living CTCs, and white arrows represent dead CTCs. L, living CTCs; D, dead CTCs. **(A)** Results of CTC drug sensitivity test in control group in vitro; **(B)** Results of CTC drug sensitivity test in PTH group in vitro; **(C)** Results of CTC drug sensitivity test in NCb group in vitro; **(D)** Results of CTC drug sensitivity test in NCbPH group in vitro.

### Case 2

A 44-year-old woman presented with a 2.5-cm × 2-cm left breast mass. Preoperative puncture was performed and suggested invasive ductal carcinoma of the left breast [immunohistochemistry: ER(−), PR(−), HER2(2+), Ki67(+30%)]. Her TNM stage was T2N0M0. Fluorescence *in situ* hybridization (FISH) was performed, which resulted positive. Thus, the EC*4 [pharmorubicin 90 mg/m^2^ (d1) and cyclophosphamide 600 mg/m^2^ (d1), 1/21d] plus TPH*4 [docetaxel 100 mg/m^2^ (d1), 1/21d, trastuzumab 8 mg/kg (d1) followed by 6 mg/kg (d1), and pertuzumab 840 mg (d1) followed by 420 mg (d1), 1/21d] regimen was selected for neoadjuvant chemotherapy. After eight cycles of neoadjuvant chemotherapy, the tumor shrank dramatically. Subsequently, modified radical mastectomy and sentinel lymph node biopsy were performed, and the postoperative pathological report suggested invasive ductal carcinoma [0.1 cm × 0.1 cm, Miller–Payne (MP) grade 4; immunohistochemistry: ER(−), PR(−), HER2(1+), Ki67(+70%)] and negative axillary lymph node. After surgery, she received standard targeted therapy of the PH regimen [trastuzumab 6 mg/kg (d1) plus pertuzumab 420 mg (d1), 1/21d]. However, 6 months later, CT revealed that several pulmonary nodules exhibited growth not observed previously, and she was diagnosed with lung metastasis from breast cancer (**Figure 3A**). The TPH regimen [albumin paclitaxel 260 mg/m^2^ (d1), trastuzumab 6 mg/kg (d1), and pertuzumab 420 mg (d1), 1/21d] was used according to the guidelines. After two cycles of treatment, the pulmonary nodules did not shrink significantly (**Figure 3B**). As histopathological information of the pulmonary nodules could not be obtained by puncture, the CTC drug sensitivity test was used to screen effective drugs for individualized treatment. All drugs selected for the CTC sensitivity test were based on guidelines and clinical experience. Unfortunately, the effects of the anti-HER2 therapy and the paclitaxel-based chemotherapy regimens were minimal, consistent with the fact that the patient’s pulmonary nodules did not shrink significantly (**Figure 4**; **Supplementary Figure S3**). Finally, the eribulin regimen [1.4 mg/m^2^ (d1, d8), 1/21d] showed the highest tumor cell killing rate of 60.5% and was used for individualized treatment. After four cycles of treatment, the pulmonary nodules were markedly reduced (**Figure 3C**). At present, the patient has achieved a PFS time of 11 months. The diagnostic and treatment process is shown in **Supplementary Figure S6**.

**Figure 3:**
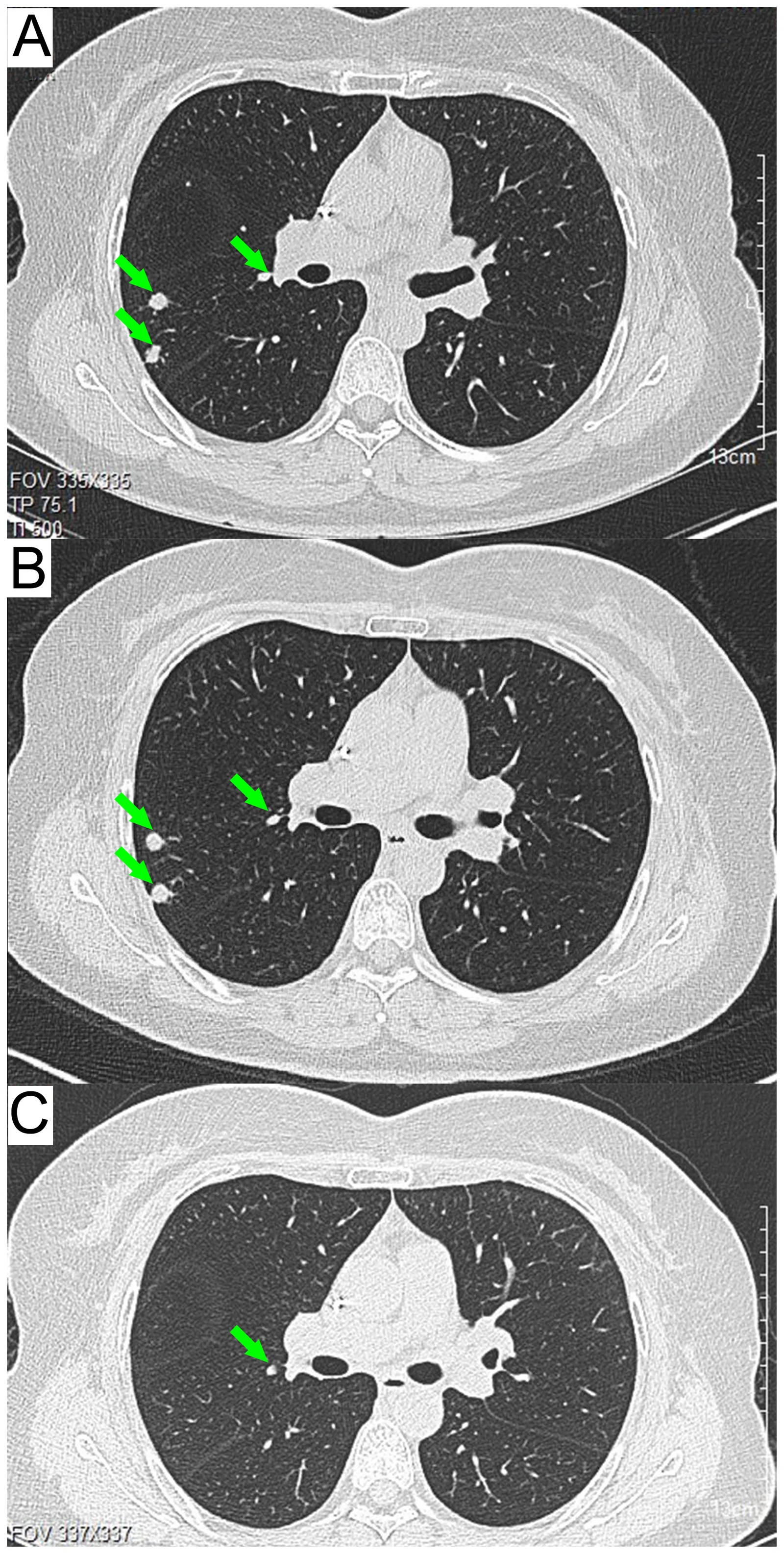
**(A)** Axial CT lung window showing multiple pulmonary nodules, which was considered pulmonary metastasis. **(B)** Axial CT lung window showing that pulmonary nodules did not shrink significantly after 2 cycles of PTH treatment; **(C)** Axial CT lung window showing that pulmonary nodules were significantly reduced after four cycles of eribulin treatment.

**Figure 4:**
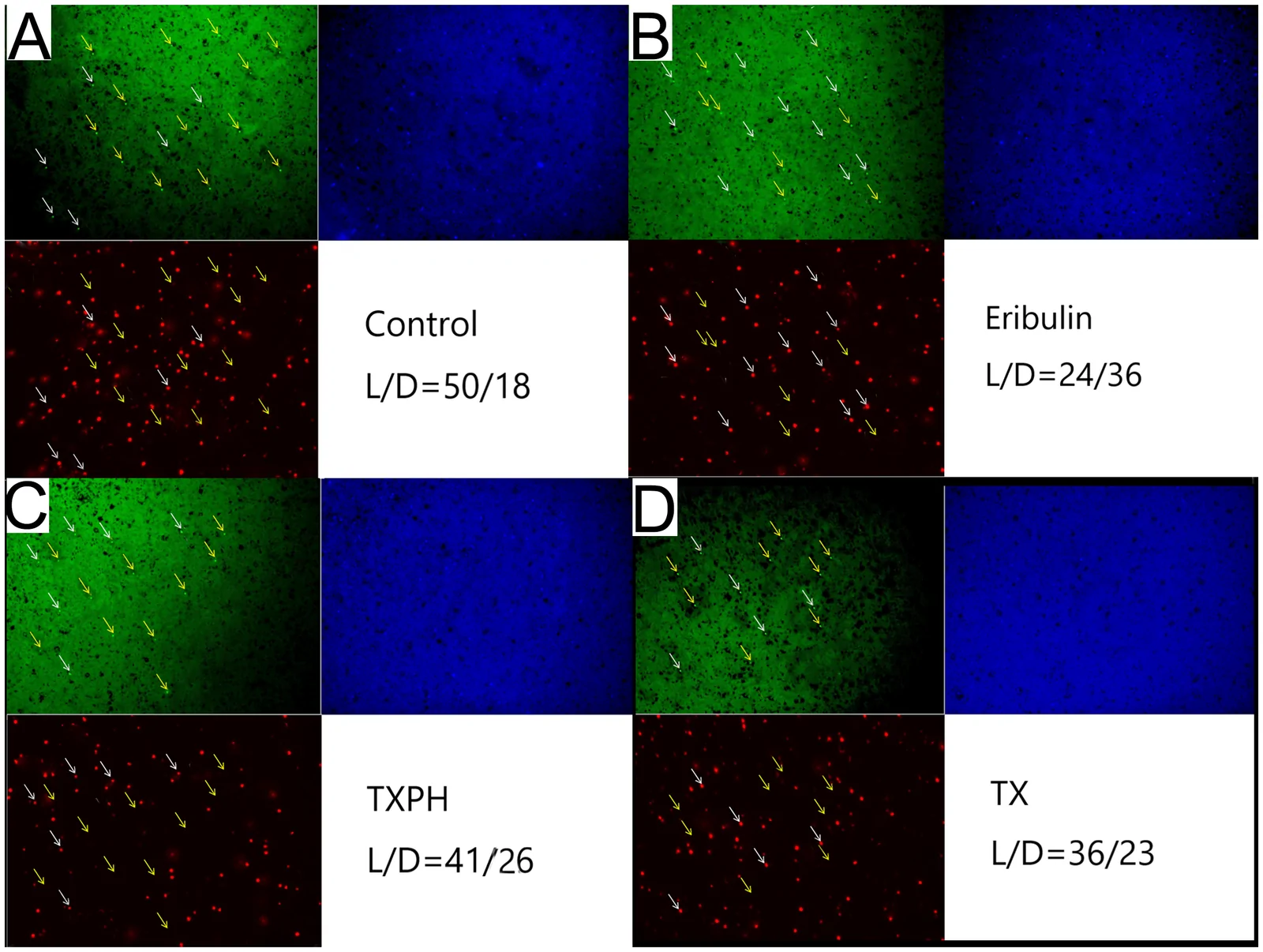
CTC drug sensitivity test results of different drugs in Case 2. Green fluorescence indicates CTCs, and red fluorescence indicates dead cells. The yellow arrows represent living CTCs, and white arrows represent dead CTCs. L: living CTCs; D: dead CTCs. **(A)** Results of the CTC drug sensitivity test in the control group in vitro; **(B)** Results of the CTC drug sensitivity test in the eribulin group in vitro; **(C)** Results of the CTC drug sensitivity test in the TXPH group in vitro; **(D)** Results of the CTC drug sensitivity test in the TX group in vitro.

## Discussion

At present, the criteria for predicting the prognosis of advanced breast cancer are mainly based on the biological phenotype of the primary tumor, the line of treatment, and the site of metastases. However, HER2 expression in the metastatic site is not necessarily consistent with the phenotype of the primary tumor (19). The process of tumor dissemination and metastasis is highly selective, and the HER2 status could change with tumor spread and metastasis (19). As a type of liquid biopsy, CTC is easy to perform and repeat compared with traditional pathological biopsies and could provide more comprehensive tumor information for individualized treatment (20, 21).

Tumor heterogeneity is one of the main reasons for advanced tumors being difficult to cure (22). On occasion, the receptor status of ER, PR, and HER2 varies between the primary tumor and the metastatic site (8). In approximately 9.5% of patients with breast cancer whose primary tumor is HER2-negative, the metastatic nodule will exhibit HER2 positivity; conversely, the metastatic nodule will exhibit HER2 negativity in approximately 21% of patients whose primary tumor is HER2-positive (23). In clinical practice, pathological information on metastases is generally needed to guide treatment. If pathological information is not available, clinicians typically resort to empirical treatment, which is often ineffective. Therefore, when pathological information cannot be accurately obtained, CTC drug sensitivity testing may be a useful tool for the prediction of the type of metastatic lesions and for the identification of effective treatments.

In the two cases we reported, both the primary tumors were HER2-positive, but with the CTC drug sensitivity test results suggesting that the lung metastases are insensitive to anti-HER2 therapy. The histopathological findings from the biopsy of the pulmonary nodules in case 1 confirmed that the patient’s HER2 status had changed from positive in the primary tumor to negative in the lung metastasis, which was consistent with the CTC drug sensitivity test results. In case 2, considering that the postoperative residual lesion was triple negative and eribulin had a very good effect, it was presumed that the HER2 status of the patient had changed to negative in the lung metastasis, although there was no conclusive evidence. In addition, the results of the CTC sensitivity test confirmed that the previously used therapeutic regimen was ineffective in the two cases, suggesting that it may have potential high value in predicting the drug resistance of tumors. Furthermore, our case report is the first to evaluate the application value of CTC drug sensitivity tests in individualized treatments of advanced breast cancer. Both patients achieved remarkable therapeutic effects, suggesting that the CTC drug sensitivity test has good reference value in guiding personalized treatment. The greatest challenge in CTC detection is the relative scarcity of CTCs (24). It is extremely difficult to capture and amplify target CTCs among a large number of normal peripheral blood cells (24, 25). Thus, several techniques have been explored to achieve maximum CTC enrichment (26, 27). In our study, we increased the collection rate of CTCs through the MACS method (28). Consequently, the CTC enrichment and drug sensitivity tests were successfully completed in two cases.

According to the 2021 National Comprehensive Cancer Network (NCCN) Breast Cancer Guidelines, the preferred first-line regimen for the two reported patients at the time of advanced metastatic presentation is still TPH therapy (31). The CLEOPATRA study confirmed that the dual-target combined chemotherapy regimen extended the median OS of the high-tumor burden population to 57.1 months, which was 16.3 months longer than that of the trastuzumab monotherapy cohort. As the two patients showed continuous disease progression after TPH treatment, we performed a CTC drug sensitivity test to screen the effective drugs for individualized treatment. All drugs used for the CTC sensitivity test were selected based on guideline recommendations and the clinical experience of senior oncologists. In case 1, biopsy of the pulmonary metastatic lesion revealed low HER2 expression (immunohistochemistry, 1+). Nevertheless, the CTC drug sensitivity test and the subsequent efficacy validation confirmed that the PH regimen maintained significant clinical effectiveness. Ultimately, a patient-centered, evidence-based personalized medication regimen was formulated for this patient.

This study has some limitations. First, many of the patients with breast cancer did not have enough CTCs to be stratified into groups for drug sensitivity screening. Second, the CTC amplification technique needs further improvement in the future. However, our case report further validates the feasibility and accuracy of CTC drug sensitivity testing in guiding individualized treatment of advanced breast cancer and suggests that it would have broad development prospects in the future, although further studies are needed to prove the validity and stability of CTC drug sensitivity testing for drug screening.

## Conclusion

In summary, our case report is the first to evaluate the application value of the CTC drug sensitivity test in guiding decisions regarding individualized treatment for advanced breast cancer, for which CTCs exhibited immense potential for clinical application. Furthermore, the CTC drug sensitivity test results were consistent with the histopathological results and the patients’ prior tumor resistance information, indicating that CTCs could be important tools for the prediction of the type of metastatic lesions and for the identification of effective treatments, in particular when histopathological information is not available.

## Data Availability

The original contributions presented in the study are included in the article/**Supplementary Material**. Further inquiries can be directed to the corresponding author.
